# Stack-Layer Dual-Element Ultrasonic Transducer for Broadband Functional Photoacoustic Tomography

**DOI:** 10.3389/fbioe.2021.786376

**Published:** 2021-10-28

**Authors:** Xiaofei Luo, Yiqi Cai, Zeyu Chen, Han Shan, Xin Sun, Qibo Lin, Jianguo Ma, Bo Wang

**Affiliations:** ^1^ Department of Biomedical Engineering, School of Basic Medical Science, Central South University, Changsha, China; ^2^ School of Instrumentation and Optoelectronics Engineering, Beihang University, Beijing, China; ^3^ School of Mechanical and Electrical Engineering, Central South University, Changsha, China; ^4^ Beijing Advanced Innovation Center for Big Data-Based Precision Medicine, Beihang University, Beijing, China

**Keywords:** dual-element transducer, oxygen saturation, broadband, functional imaging, photoacoustic tomography (PAT)

## Abstract

Current Photoacoustic tomography (PAT) approaches are based on a single-element transducer that exhibits compromised performance in clinical imaging applications. For example, vascular, tumors are likely to have complicated shapes and optical absorptions, covering relatively wide spectra in acoustic signals. The wide ultrasonic spectra make it difficult to set the detection bandwidth optimally in advance. In this work, we propose a stack-layer dual-element ultrasonic transducer for PAT. The central frequencies of the two piezoelectric elements are 3.06 MHz (99.3% bandwidth at –6 dB) and 11.07 MHz (85.2% bandwidth at –6 dB), respectively. This transducer bridges the sensitivity capability of ultrasound and the high contrast of optical methods in functional photoacoustic tomography. The dual-element transducer enabled multiscale analysis of the vascular network in rat brains. Using a multi-wavelength imaging scheme, the blood oxygen saturation was also detected. The preliminary results showed the great potential of broad-bandwidth functional PAT on vascular network visualization. The method can also be extended to whole-body imaging of small animals, breast cancer detection, and finger joint imaging.

## Introduction

Photoacoustic imaging takes advantage of the low ultrasound scattering and high optical contrast in the biological tissues and offers cross-scale structural and functional images with excellent spatial resolutions (Ku and Wang, 2000; Wang, 2008; Moore et al., 2018; Luo et al., 2019; Zhou et al., 2019; Liu et al., 2020; Zhou et al., 2020). So far, PAT has been applied in a wide spectrum of biomedical applications (Jo et al., 2018; Oraevsky et al., 2018; Na et al., 2021). For example, by providing vascular structure (Wang et al., 2003; Wang et al., 2006; Li et al., 2015), deoxyhemoglobin (Li et al., 2018), oxyhemoglobin total hemoglobin [tHb] and blood oxygen saturation [S0_2_] information, PAT can visualize blood vessel networks in small animal brains (Yang et al., 2009; Mallidi et al., 2011; Wang and Hu, 2012; Lin et al., 2018; Mercep et al., 2018; Zhang et al., 2018) for functional biomedical diagnostics.

Although photoacoustic signals cover a wide spectral range, a single transducer receives only part of the spectrum because of its limited bandwidth (Ku and Wang, 2001; Wang and Hu, 2012). In general, a high-frequency transducer can provide a better resolution, but the signal is relatively weak due to the high ultrasound attenuation in the media. This leads to a trade-off between imaging resolution and sensitivity (Ku et al., 2004). Several approaches have been developed to counteract the limited bandwidth. For example, in order to obtain more complicated structure imaging, (Ku et al., 2004) employed multiple ultrasonic transducers with various central frequencies simultaneous. However, using different transducers involved a complex assembly process, which is hard to guarantee signals in the same rotation phase. Some groups have developed a dual-element transducer for the detection of the distribution and reconstruction of the lipid in tissues. However, due to the mismatch of the two acoustic fields in the transducer, the obtained signals are not spatially coincident with each other (Cao et al., 2020). An alternative approach by using an ultra-broadband transducer and tomographic reconstruction was designed to obtain both high-frequency and low-frequency information at the same time (Aguirre et al., 2017; Haedicke et al., 2020). However, in theory, the sensitivity of this approach is lower compared to a dual-element ultrasound transducer. Although PVDF based transducers and optical-ultrasound detection methods have a wide-spectrum response (Xiao et al., 2016; Sappati and Bhadra, 2018; Wissmeyer et al., 2018; Shepelin et al., 2019), (as shown in Table 1), The deficiency of these methods was that they are weak in or lack the ability to produce ultrasound for simultaneous photoacoustic and ultrasound imaging. Therefore, there is still a strong demand for developing a new method to overcome the limited bandwidth for photoacoustic imaging. Stack-layer dual-frequency transducers with large band gaps have been developed previously for super-harmonic imaging, which is not suitable for sensing broadband photoacoustic signals. Nevertheless, similar designs with stack-layer dual-element transducers hold the potential in photoacoustic imaging due to the broadband coverage.

**TABLE 1:** Comparison of optical, piezoelectric, PVDF (polymer) and our dual-element piezoflex PZT (composite materials) ultrasound transducer.

Detector type	Sensitivity Nep (mPa/Hz^1/2^)	d33 (pC/N)	BW (%)
Piezoelectric	0.2	410	60–80
Optical	78	—	90
PVDF	14.4	13–28	163.6
PZT-5H 1–3 composite	7.1	550	99.3

Here, we designed and fabricated a stack-layer dual-element ultrasound transducer with central frequencies of 3.06 and 11.7 MHz. This broadband ultrasound transducer can directly obtain high-frequency signals and low-frequency signals simultaneously, enabling multiscale imaging of targeted regions. The imaging of phantom samples and rat brains was successfully conducted to verify the adaptability and capability of our method.

## Materials and Methods

### Design of Stack-Layer Dual-Element Transducer

Stack-layer dual-element ultrasonic transducer is proposed to cover a broad bandwidth at a coincident position in the acoustic field. First, two active elements, i.e., two piezoelectric layers, were used to cover two spectral ranges, which overcame the intrinsic bandwidth limitations of piezoelectric materials. Second, the high-frequency element was in front of the low-frequency one, which formed a stack-layer arrangement spatially. The stack-layer arrangement of the two elements assures the overlap of the two beams and enables broadband coverage within the beam.

**FIGURE 1:**
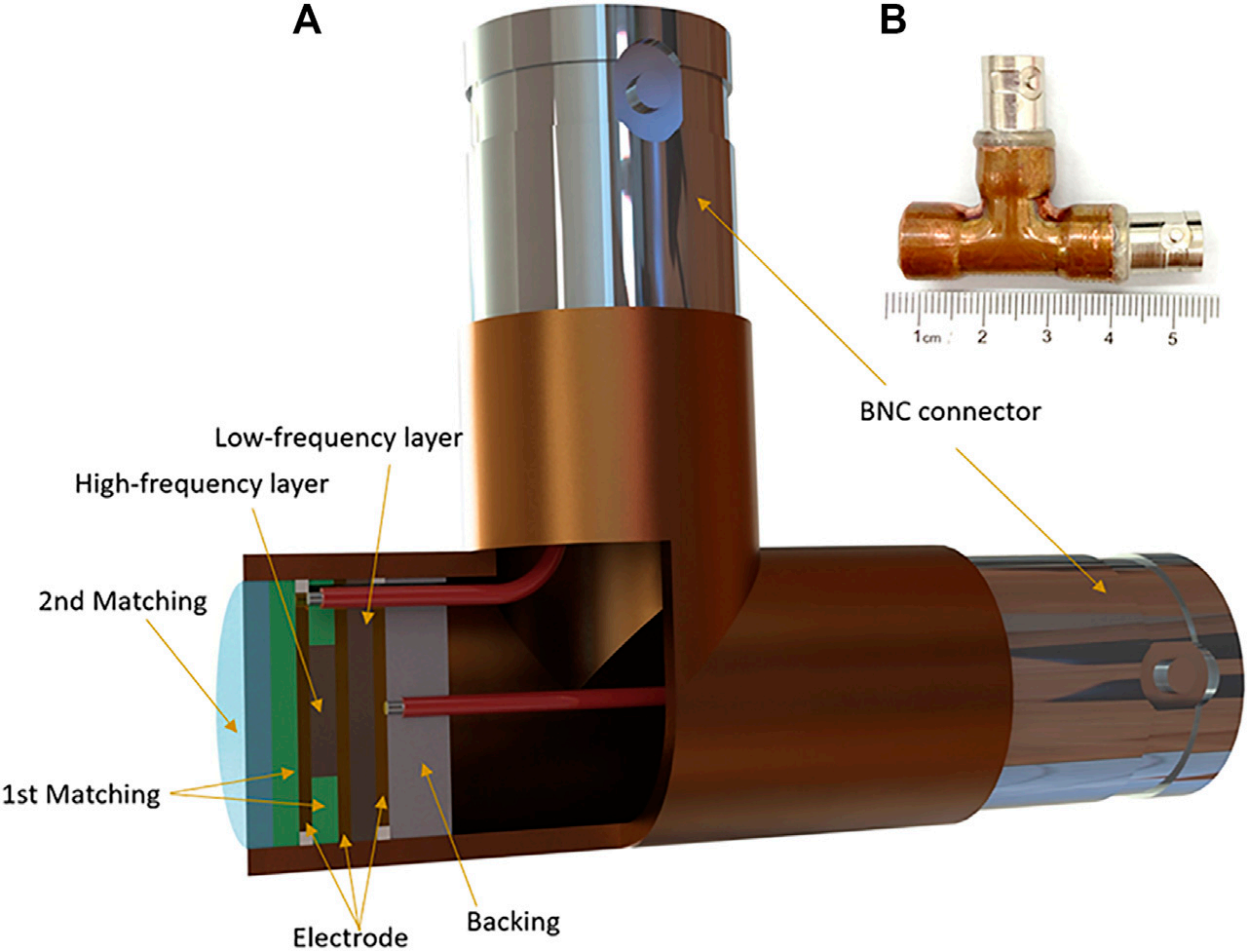
Dual-element planar transducer. **(A)** The cross-sectional structure of the dual-element transducer. **(B)** Photograph of the transducer.

The cross-sectional structure of the dual-element transducer and photograph of the prototype for PAT imaging are shown in Figures 1A,B. The outer diameter of the brass transducer is 12 mm. The piezoelectric elements are made of PZT-5H 1–3 composite, and the aperture is 7 mm × 5 mm and 4 mm × 4 mm for the low and high-frequency elements, respectively. The aperture difference of the two elements was due to the need for electrical impedence matching to the input impedence of the data acquisition system of 50 Ω. Because piezoelectric elements with higher frequencies have smaller electrical impedence. To balance the two elements, the aperture of the high-frequency element needs to be smaller. Gold was sputtered on both surfaces of the piezoelectric layers as electrodes. Tungsten powder mixed with epoxy resin (EPO-TEK 301) was centrifuged and applied to the ceramic as the backing layer. A mixture of alumina powder and epoxy was used for the first matching layer, and pure epoxy was used as the second matching layer. Properties of piezoelectric materials, acoustic matching layers, and backing layer used for the dual-element transducer are shown in Table 2.

**TABLE 2:** Material properties and dimensions for the dual-element transducer.

Parameters	Active low freq	Active high freq	1st mating	2nd mating	Backing
Density (g/cm^3^)	4.1	4.1	1.865	1.13	6.237
Velocity (km/s)	3.2	3.2	2.923	2.556	1.087
Acoustic impedance (MRayl)	13.1	13.1	5.5	2.9	11.3
Thinkness (μm)	533	160	56	36	10,000

### Transducer Characterization

**FIGURE 2:**
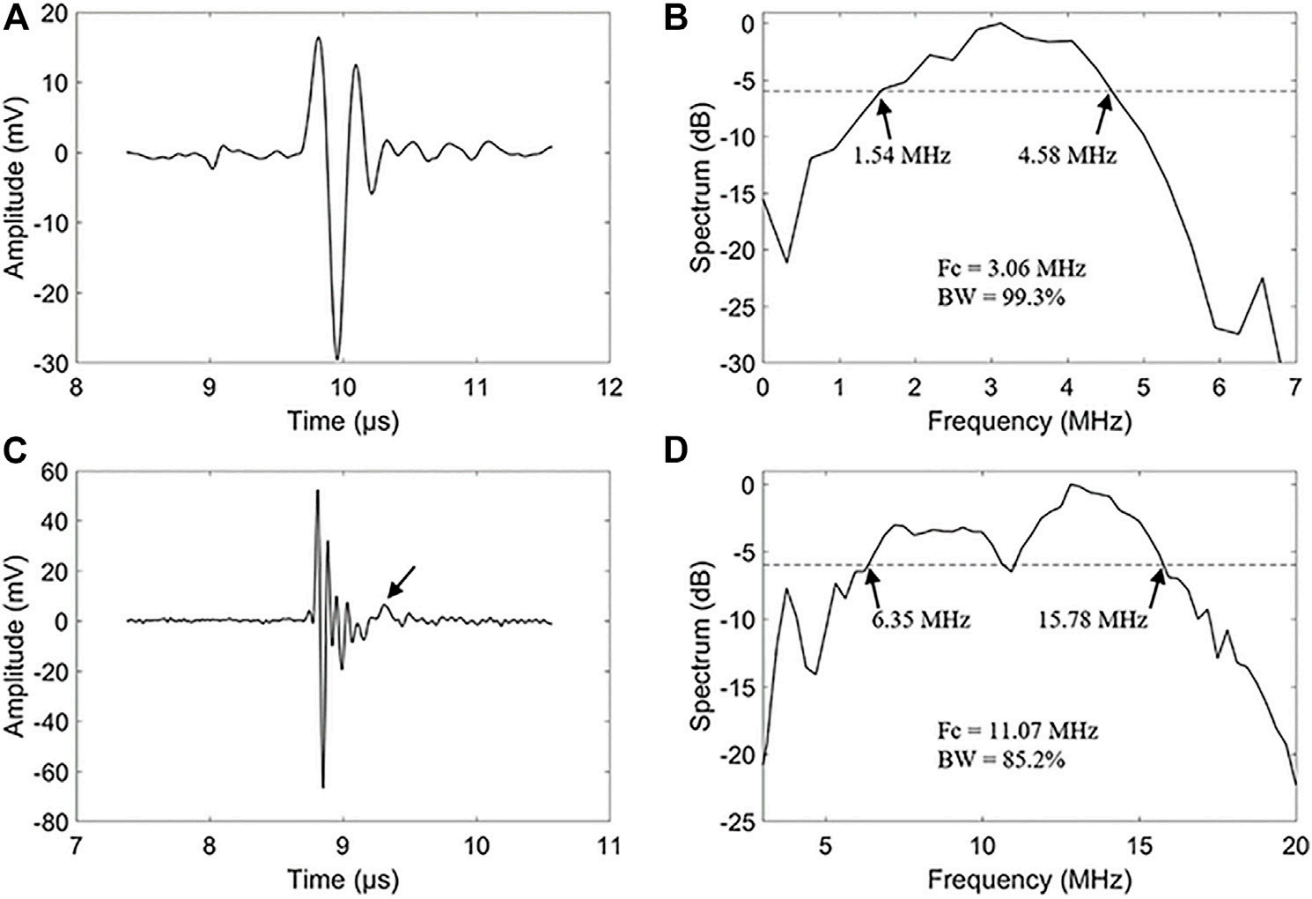
Transmit characteristic of the dual-element transducer. **(A)** A hydrophone measured waveform of the 3.06 MHz frequencies element. **(B)** Spectrum diagram of the 3.06 MHz frequencies element of the dual-element transducer. **(C)** A hydrophone measured waveform of the 11.07 MHz frequencies element. **(D)** Spectrum diagram of the 11.07 MHz frequencies element of the dual-element transducer.

The impulse response waveforms of the 3.06 and 11.07 MHz transducer elements were characterized by a needle hydrophone (HGL-0200, Onda Corp, Sunnyvale, CA, United States). In the measurements, electrical pulses from a function generator (UTG2062B, Uni-Trend Technology Co., Guangdong, China) excited the transducer elements and signals from the needle hydrophone were recorded by a digital oscilloscope (MSO54, Tektronix, Inc., Beaverton, OR, United States). To ensure the measurement accuracy, the hydrophone was placed coaxially with the dual-element transducer in a deionized water tank and scanned along the axial direction of the transducer to find the waveform with the maximum peak-to-peak voltage. We compared the time-domain waveforms and their corresponding frequency spectra. The transmission responses of the 3.06 MHz element are shown in Figures 2A,B. The –6 dB bandwidth covers from 1.54 to 4.58 MHz, corresponding to a fractional bandwidth of 99.3%. Figures 2C,D show the transmission responses of the 11.07 MHz element, leading to a –6 dB bandwidth coverage of 6.35–15.78 MHz and a fractional bandwidth of 85.2%.

It’s noticed that there are some alias echos in the high-frequency signal, as noted with an arrow in Figure 2C. The inconsistency of the acoustic impedance between the backing and the piezoelectric material is the main cause of aliasing echo. We glued the backing to the piezoelectric layer with a low-impedance epoxy resin, which also resulted in the reduction of the equivalent acoustic impedance. The reduction of the acoustic impedance of the backing layer results in the reflection of high-frequency echoes at the backing, resulting in some aliasing echoes.

### Experiment Setup

**FIGURE 3:**
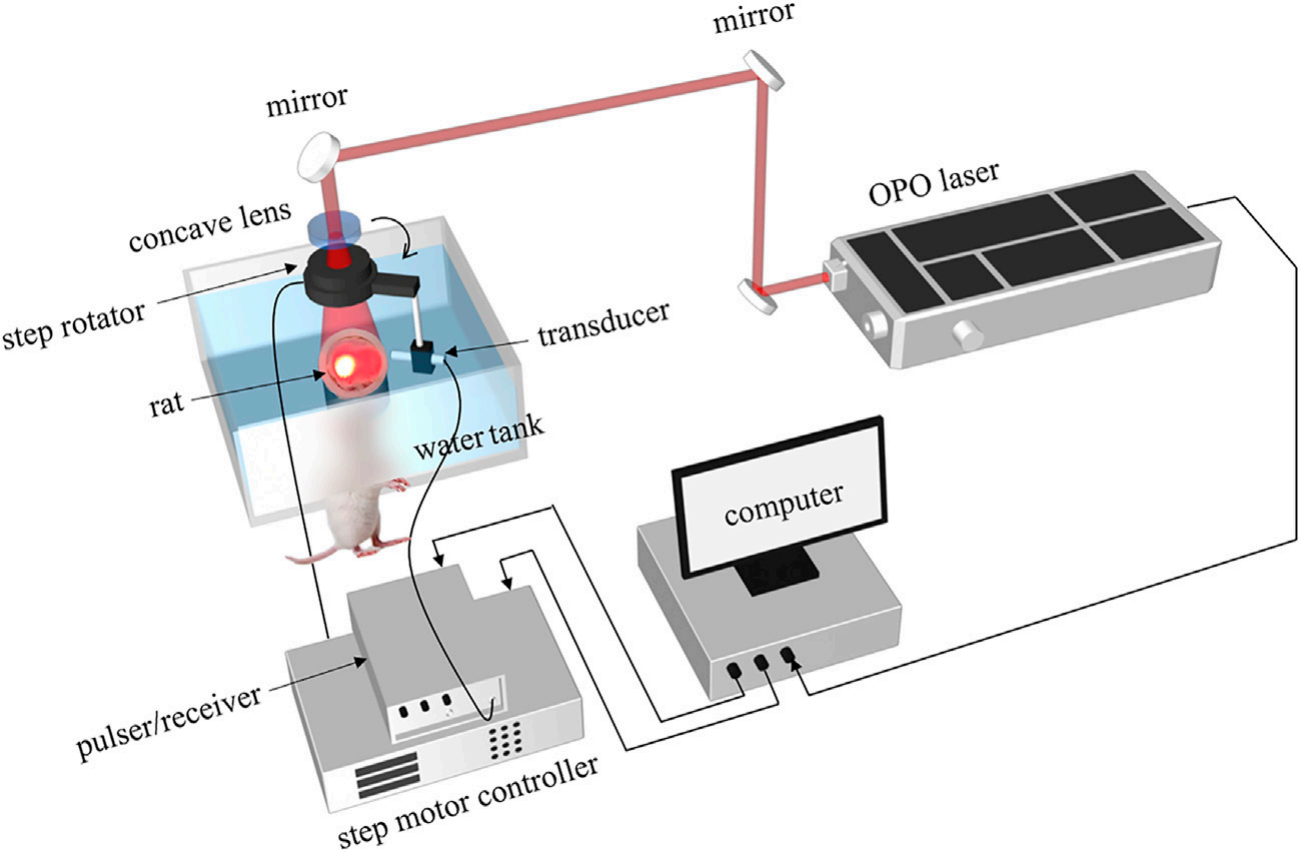
Schematic of the 2D circular-scanning-based dual-frequency PAT system.

The photoacoustic sensing capability of the dual-element transducer was evaluated on a leaf phantom and a rat brain experimentally. First, to test our transducer, one piece of leaf veins with ∼8 mm dimension was used as a phantom, which was placed on a table with a diameter of 3 cm. Second, the vasculature of the rat brain was used to demonstrate the imaging performance. Both imagings share the same experimental setup with a two-dimensional (2D) scanning. The schematic of the 2D PAT system was shown in Figure 3. The target was illuminated by using a pulsed laser from an optical parametric oscillator (OPO) laser (SpitLight 600 OPO-532 mid band, Innolas). The optical intensity on the top of the phantom was about 5 mJ/cm^2^, and the repetition rate was 20 Hz. The total step number of the 2D scan was 360 with an angular step of 1°. The distance between the rotation center to the transducer detection surface was about 30 mm. The signal was first amplified by a pulser/receiver (DPR500, Ultrasonics), and then digitized with an acquisition card (NI-5124, 12 bit, 100 MHz sampling frequency) in the computer. The whole system was synchronized with the laser. And the data were collected from the hard disk for later processing.

### Animal Protocol

We performed functional PAT of rat brains *in vivo* to test the capability of our dual-element transducer and assessed the oxygenation level of hemoglobin and [tHb] level simultaneously. Sprague Dawley rats (∼60 g, 3 weeks, Hunan SJA laboratory animal Co., LTD.) were used. The protocol of animal experiments has been approved by the animal ethical committee of the Central South University of China. The hair on the head of the rat was removed using hair remover cream before imaging. The rat was anesthetized with Pentobarbital [120 mg/kg, Intra-peritoneal (IP)] and kept motionless throughout the experiment. A homemade animal holder was used to fix the rat head. And a transparent membrane between water and rat head was used to seal the cylindrical hole.

According to the absorption difference between hemoglobin and tissue, a pulse laser with a wavelength of 760 and 840 nm is used to image the vascular morphology, total hemoglobin, and oxygen saturation distribution. This enables us to achieve multi-parameter photoacoustic tomography.

In order to obtain the photoacoustic signal for each image, the dual-element transducer scanned 360 steps in the horizontal plane around the cerebral cortex at the back of the brain with 1° per step. For signal averaging, four laser pulses were applied at each scanning position. The signal acquisition period of each imaging was about 10 min. In total, we obtained four images corresponding to two frequencies at two wavelengths. After collection of data for imaging, the rat recovered normally and no obvious health problems were observed. In the end, the rat was sacrificed using pentobarbital.

### Data Processing

PAT acquisitions with dual-element transducers were conducted to image the phantom. And PAT acquisitions with 760 and 840 nm laser wavelengths were performed to image the rat brain. Then, the Hilbert transform was applied to the signals, and the resulted complex data was employed for the reconstruction of two-dimensional PAT images with a conventional back-projection method (Xu and Wang, 2005). The final reconstructed images were presented by the amplitudes of the pixel values. In this work, we also merged 3.06 MHz element images and 11.07 MHz element images to display the morphology and distribution of the rat brain and its surrounding tissues. For the merged 2D PAT images, the equation is used to calculate pixel values in the hue, saturation and value (HSV) color model (Wang et al., 2021).

$$\begin{array}{l} h=2/\left(3\pi \right)\times angle\left(A_{3.06}+iA_{11.07}\right)\\ s=1\\ v=\mathrm{mod}ulus\left(A_{3.06}+iA_{11.07}\right) \end{array}$$

Here, *h*, *s*, and *v* represent the hue, saturation, value components of the pixel, $A_{3.06}$ , $A_{11.07}$ are the normalized photoacoustic image acquired by 3.06 MHz frequency element and 11.07 MHz frequency element, i is the imaginary unit.

## Results

### Phantom Results

**FIGURE 4:**
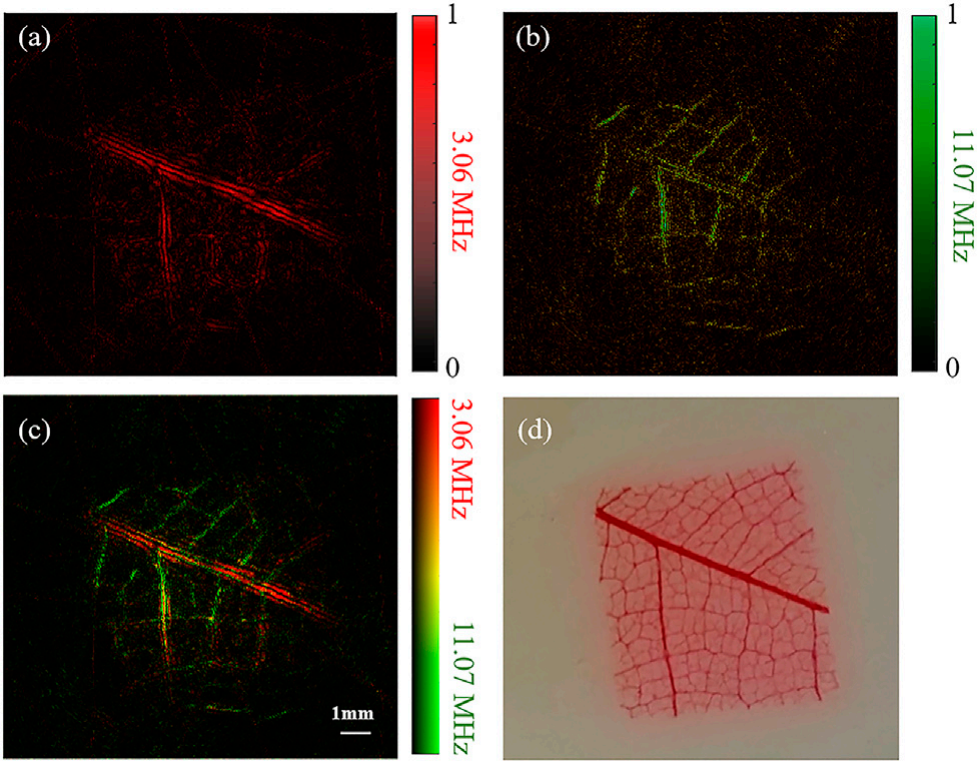
Reconstruction results of a piece of leaf veins using a dual-element planar transducer. **(A–C)** The results given by the low-frequency element, the high-frequency element and the merged results of the two elements. **(D)** Photograph of the leaf veins.

Photoacoustic images of a piece of leaf veins obtained by the dual-element planar transducer are shown in Figure 4. Figures 4A,B are the reconstruction PAT images employed by the 3.06 MHz element and 11.07 MHz element, respectively. A merged image of Figures 4A,B is shown in Figure 4C. A photograph of the piece of leaf veins shown in Figure 4D was used for comparison.

The color pixel in Figure 4C represents the relative intensities of the 3.06 and 11.07 MHz frequencies photoacoustic signals. It becomes purely red if only 3.06 MHz photoacoustic signals are generated, and a purely green pixel means that only 11.07 MHz photoacoustic signals are received. The overall signal intensity is represented by the brightness of the pixel. As shown in Figure 4A, the 3.06 MHz element has a better imaging effect on the main vein branches but has a poor reconstruction effect on the small branches. On the contrary, the 11.07 MHz element image the small veins clearly with a compromised performance at the main veins. Therefore, with the benefit of our dual-element transducer, the broadband signal can be obtained simultaneously at one scan without changing the rotation phase. This has been indicated with the merged reconstruction image in Figure 4C, which is similar to the photograph of the leaf veins shown in Figure 4D.

### Animal Results

**FIGURE 5:**
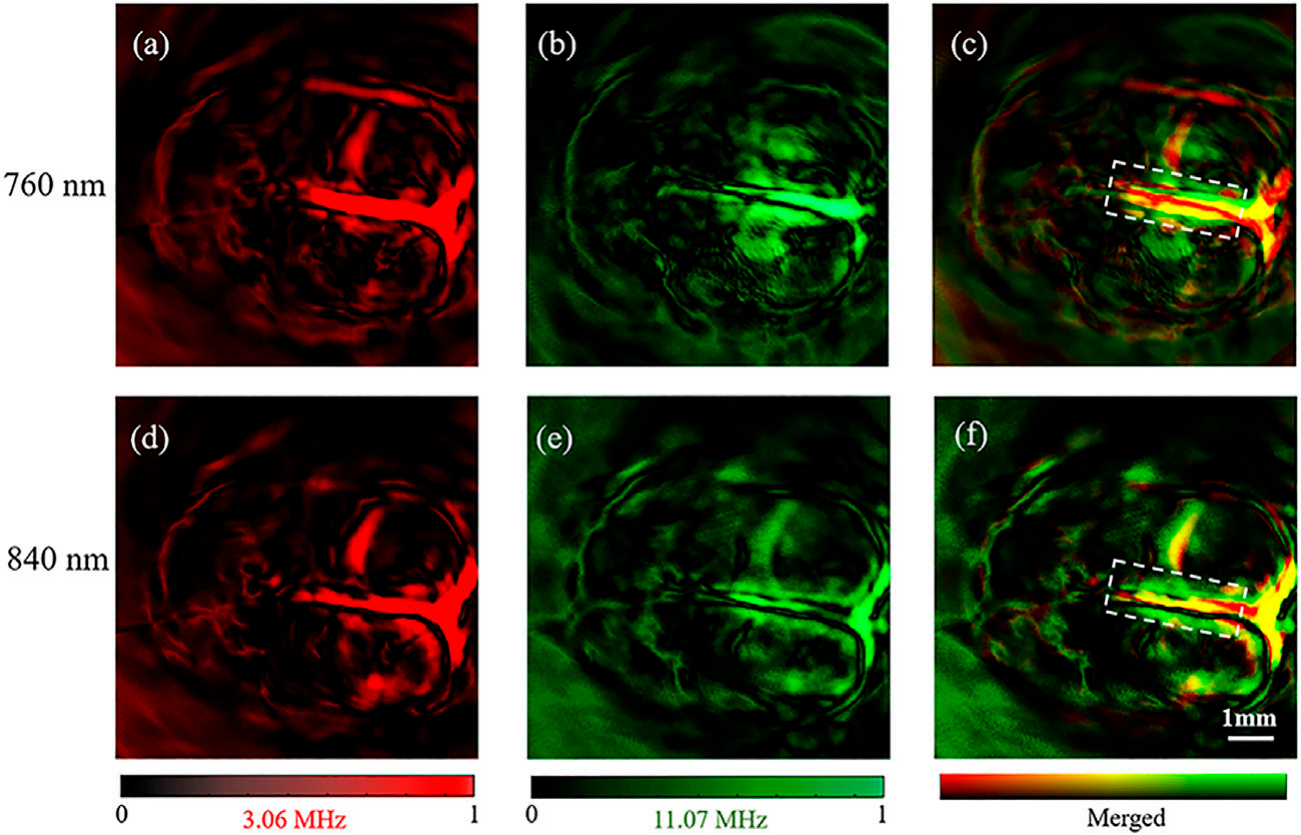
Reconstruction results of rat brain. **(A–C)** were the images obtained by using the 3.06 MHz element, the 11.07 MHz element and the merged image of **(A,B)** at 760 nm OPO laser, respectively. **(D**–**F)** were the images obtained by using the 3.06 MHz element, the 11.07 MHz element and the merged image of **(D,E)** at 840 nm OPO laser, respectively.

By application of our transducer, we obtained the results of photoacoustic reconstruction of the rat cortex. Results given by the 3.06 MHz element, the 11.07 MHz element, and the merged signals at the 760 nm laser are shown in Figures 5A–C, respectively. Similarly, Figures 5D–F show the set of the three images excited by the 840 nm laser. Since tissues have different absorption characteristics under various light excitations, the PAT images of the two wavelengths show different structural characteristics. Images with 760 nm laser excitation mainly show the distribution of deoxyhemoglobin, whereas those with 840 nm laser excitation mainly show the distribution of oxygenated hemoglobin.

The brain images obtained under the 760 and 840 nm laser present the same vascular structure but different magnitudes of optical absorption. With high absorption contrast between the blood and background brain tissue, all of the brain images show the superior sagittal sinus and some branches can be clearly observed and match well with the vascular.

Similar to the leaf veins results, we obtained a good imaging effect on the main vascular branches but a poor effect on the small vascular by the 3.06 MHz element with both 760 and 840 nm of optical excitation (Figures 5A,B). In comparison, the small vascular, but not the main vascular, was observed by the 11.07 MHz element with the two excitations (Figures 5B–E). Therefore, our results further confirmed that the dual-element transducer obtained the 11.07 MHz and the 3.06 MHz information at the same time without changing the rotation phase.

**FIGURE 6:**
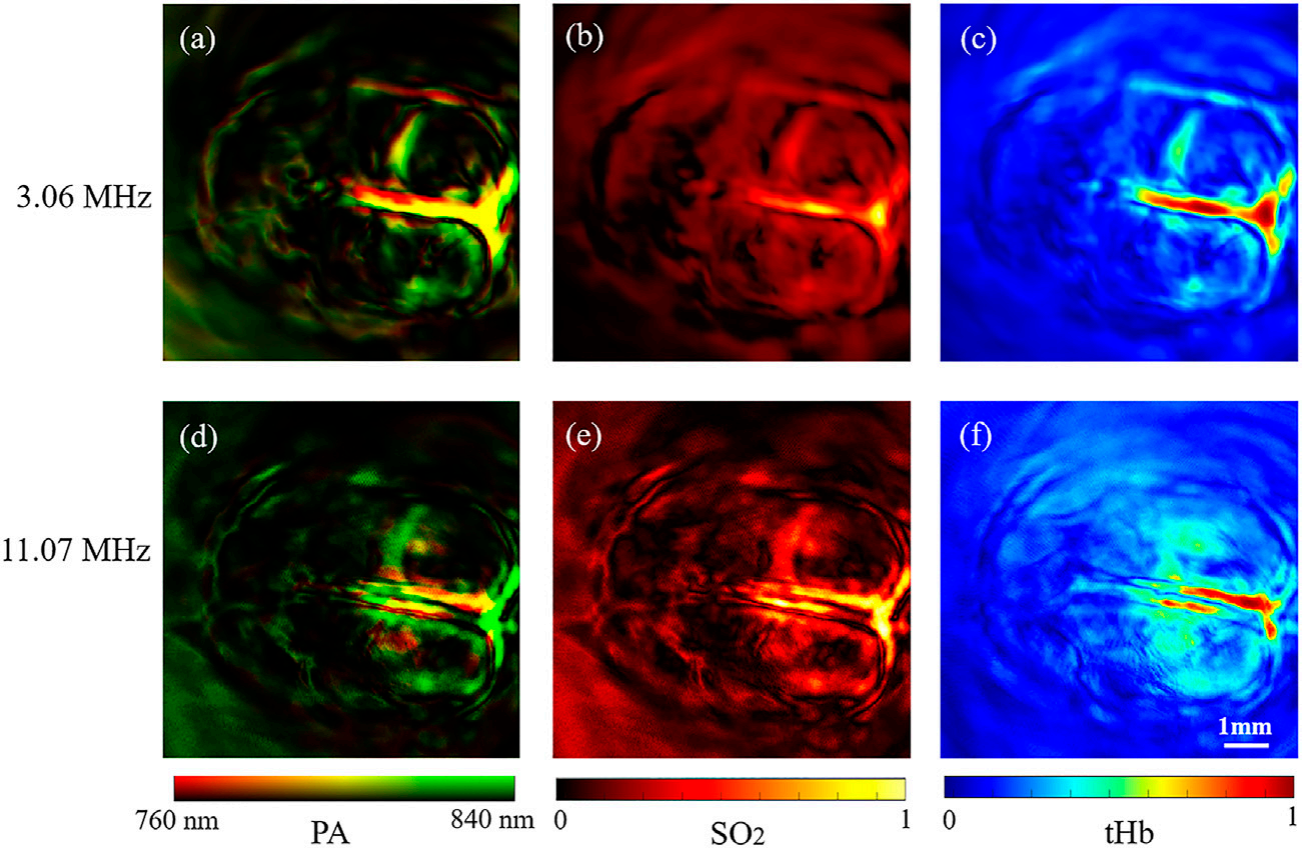
Functional 2D PAT results of rat brain. **(A**–**C)** Dual-wavelength structure, [tHb] and [SO2] results by 3.06 MHz frequency element of the rat brain. **(D**–**F)** Dual-wavelength structure, [tHb] and [SO_2_] results by 11.07 MHz frequency element of the rat brain.

For further investigation, we calculated the [tHb] and [SO_2_] distribution of the rat brain [10]. We selected the region around the superior sagittal sinus as the region of interest. Results showed that the levels of [SO_2_] and [tHb] in this region were significantly higher than that of other branches in Figure 6.

## Discussion

In PAT systems, the “limited bandwidth” is one of the prime problems of the current existing ultrasonic transducers, which has not been effectively solved (Awasthi et al., 2020). The limited bandwidth of transducers may cause many problems of PAT such as feature loss, low sensitivity and limited resolution (Wang et al., 2021). This prevented the acquisition of high-quality photoacoustic images. The goal of this work is to find an effective way to solve this problem in terms of the device. Therefore, we designed and fabricated a stack-layer dual-element ultrasound transducer. Compared with the traditional single element transducer, our transducer obtains low frequency (3.06 MHz, 99.3% bandwidth at –6 dB) and high-frequency information (11.07 MHz, 85.2% bandwidth at –6 dB) in one scan.

*In vivo*, PAT of rat brian results showed the structure of 10 mm × 10 mm region with brain hemoglobin and oxygen saturation distribution. The functional results obtained by the 3.06 MHz element were compared with those obtained by the 11.07 MHz element (Figure 6). The 3.06 MHz element imaged the main blood vessels, and the 11.07 MHz element clearly imaged small blood vessels with tissue information. The dual-elements transducer with 3.06 and 11.07 MHz elements formed complementary information in merged photoacoustic images (Figures 6C,F). The stacked design of the transducer enables one scan to obtain high-frequency and low-frequency information at the same rotation phase simultaneously, which avoids the physiological changes caused by different scans and transducers. In addition, dual-frequency elements transducer supplied different depth information through dual-wavelength systems. It’s noted that in this study, we used 760 nm wavelength and 840 nm wavelength to obtain the oxygen saturation, while the light absorption peak of a blood vessel is around 520 nm wavelength. Thus, in our results, the blood vessel density is not as high as those obtained around 532 nm. In future studies, brain blood vessel imaging with a 532 nm laser can be performed with our system.

However, there are still some limitations and remaining challenges for future advances. Firstly, our low-frequency element is at 3.06 MHz. To perfectly image the brain, a system with a lower central frequency (for example, 1 MHz) is needed. Because the skull-induced acoustic attenuation is frequency-dependent, the transcranial PA signal is centered at ∼ 0.75 MHz (Na et al., 2020). To see a more subtle vascular structure, a central frequency higher than 11.07 MHz would be preferable (Xu and Wang, 2003). Second, when the radiation exposure reaches the ANSI limit, the signal to noise ratio increased by about three times.

In this work, the advantages of the dual-frequency ultrasonic structure are preliminarily verified. The next step is to make an array for three-dimensional imaging. In addition, to obtain comprehensive structural information of the vascular networks by utilizing the ultra-wide bandwidth of the transducer, we can attach an acoustic lens to our dual-element transducer for focusing detection in photoacoustic microscopy (Xiao et al., 2016).

## Conclusion

In summary, a stack-layer dual-element transducer with 3.06 MHz/11.07 MHz central frequencies was designed and fabricated to improve the bandwidth coverage. The 3.06 MHz low-frequency transducer element provides enhanced photoacoustic sensitivity, while the 11.07 MHz high-frequency transducer element maintains excellent spatial resolutions for high-resolution imaging. This transducer was employed for highly sensitive detection and precise localization of rat brain vascular.

Compared with the conventional single-element transducer, this dual-element transducer acquired a broadband signal for complicated targets effectively. We tested our transducer with both phantom and animal experiments. We also demonstrated that the stack-layer dual-element transducer boosted the capability of the PAT system, enabled multiscale analysis of the vascular network in rat brains, and realized the evaluation of blood oxygen saturation with a multi-wavelength imaging scheme. The advantages of the dual-frequency ultrasonic structure are preliminarily verified. The next step is to make an array for three-dimensional imaging. Different from most existing methods for improving the bandwidth, this dual-element transducer features simplicity in implementation and can be easily adapted to most current PAT systems. Thus it has a great potential for vascular network visualization, small-animal whole-body imaging, and cancer detection.

## Data Availability

The raw data supporting the conclusions of this article will be made available by the authors, without undue reservation.
